# Complete and Fast Recovery from Idiopathic Facial Paralysis Using Laser-Photobiomodulation

**DOI:** 10.1155/2020/9867693

**Published:** 2020-03-11

**Authors:** João Paulo Colesanti Tanganeli, Simone Saldanha Ignácio de Oliveira, Tamiris da Silva, Kristianne Porta Santos Fernandes, Lara Jansiski Motta, Sandra Kalil Bussadori

**Affiliations:** ^1^Post-Doctoral Student Program in Biophotonics Applied to Health Sciences, University Nove de Julho, SP, Brazil; ^2^PhD University of São Paulo, SP, Brazil; ^3^Post-Graduate Program in Biophotonics Applied to Health Sciences, University Nove de Julho, SP, Brazil; ^4^PhD Biophotonics Applied to Health Sciences, University Nove de Julho, SP, Brazil

## Abstract

Idiopathic facial paralysis, also known as Bell's palsy, exerts a negative effect on the quality of life. Although the prognosis is good in the majority of cases, a significant percentage of affected individuals may have sequelae that can negatively affect their lives. The use of therapeutic measures as early as possible can improve the prognosis. This article describes the successful use of laser-photobiomodulation as a single therapy in a patient with Bell's palsy and confirms the possibility of using this therapeutic modality as a good choice, since it is a therapy that is painless, comfortable, and without systemic side effects. The findings demonstrate that the adequate use of laser-photobiomodulation can be an effective therapeutic option for patients with Bell's palsy, regardless of the age, shortening the recovery time obtained with conventional therapies and avoiding sequelae. Further studies are needed for the establishment of adequate protocols.

## 1. Introduction

Idiopathic facial paralysis, also known as Bell's palsy, is the most common form of facial paralysis, accounting for 60 to 75% of cases [1]. This condition affects the seventh cranial nerve and is characterized by acute, unilateral onset that compromises function and esthetics, exerting a considerable impact on the social, professional, and psychological aspects of the lives of affected individuals [2].

The etiology of this condition remains unclear [3–5]. However, some aspects are considered possible triggers, such as genetic factors, viral infection, autoimmune disease, diabetes mellitus, pregnancy, Lyme disease, emotional factors, and stress [2, 3, 5, 6]. Moreover, recent studies point to the reactivation of herpes simplex virus and/or herpes zoster (shingles) as possible triggers for the onset of paralysis [3, 5, 7].

The diagnosis can be obtained through clinical examination, but complementary exams can be useful, especially for the elucidation of the differential diagnosis in relation to certain diseases, such as Ramsay Hunt syndrome [3, 8], Lyme disease [9], and lymphoma [10].

The incidence of Bell's palsy is between 23 and 35 cases for every 100,000 people [2]. The most common age group for the onset of facial paralysis is between 30 and 50 years [2], but children can be affected, which is a situation that deserves an even more detailed investigation [11–13]. The initial symptoms include the appearance of an earache several days prior to the onset of the paralysis that requires analgesia [1]. The condition ranges from mild to complete paralysis of the affected side of the face [1, 3, 4].

Different treatments have been proposed to achieve rapid recovery without significant sequelae. Such treatments include botulinum toxin [14], acupuncture, kinesio taping [15], facial expression exercises [2, 3], corticoids, antiviral drugs, electrical stimulation [3], and laser-photobiomodulation (L-PBM) [2, 4, 12, 16].

The occurrence of Bell's palsy during pregnancy [17] requires integrated follow-up with the prenatal medical team. Individuals with diabetes can suffer unusual consequences, such as hearing loss [6, 18]. Permanent speech difficulties [19] and an association with temporomandibular disorders have also been described [16].

Some studies have been conducted to predict the prognosis and quantify the degree of impairment in Bell's palsy [5, 20]. Although the prognosis is good, with complete or adequate recovery in 70 to 85% of cases [1, 3], shortening the recovery time is a common concern for both affected individuals and the health professionals in charge of treating this condition. Indeed, the early institution of measures to accelerate the recovery process leads to better results [1–4, 16]. Laser-photobiomodulation administered with adequate protocols by trained professionals has proven to be a good option for accelerating the recovery process in both adults and children [2, 4, 12, 16].

## 2. Case Presentation

**Figure 1:**
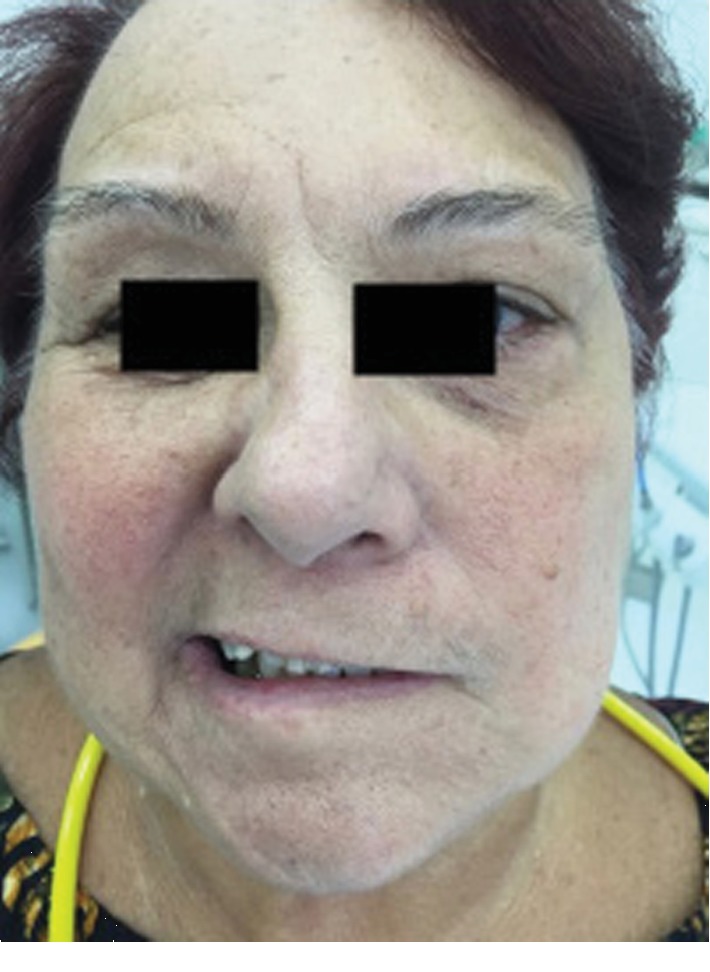
Patient with Bell's palsy—pretreatment.

**Figure 2:**
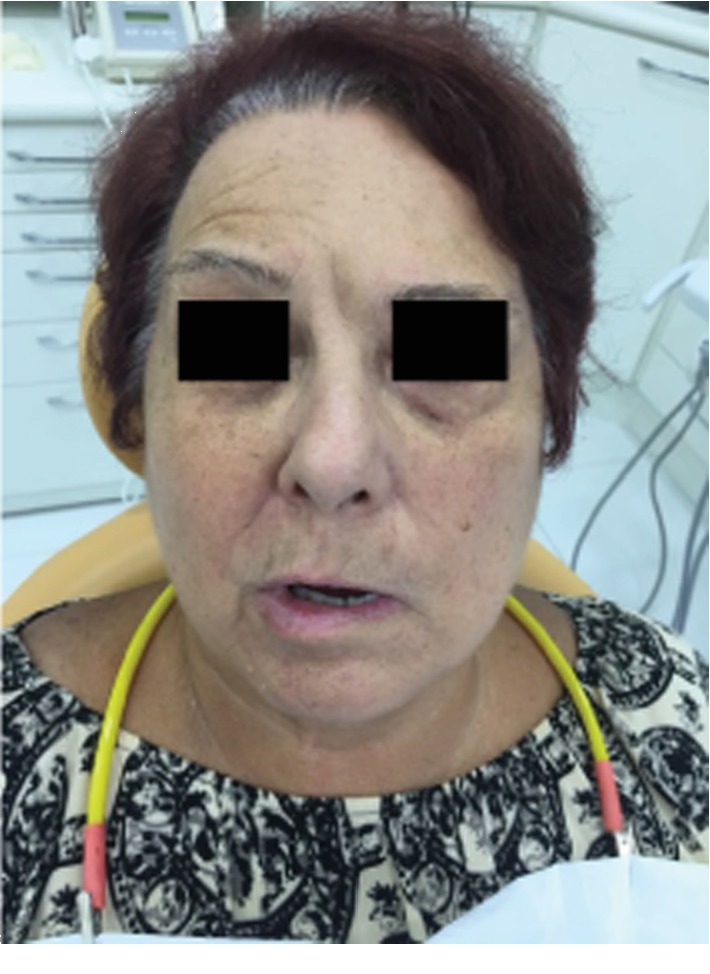
Patient with Bell's palsy—pretreatment.

AMFC, a 71-year-old female patient, presented with a sudden onset of unilateral facial paralysis preceded by pain in the masticatory muscles (masseter and temporal). She sought an emergency service on March 4^th^, 2018. After cranial tomography for the differential diagnosis in relation to a possible transitory stroke, the neurologist diagnostic was Bell's palsy. In our first evaluation (Figures 1 and 2), we classified the case as grade V (severe), according to the House-Brackmann Scale (Table 1). This classification will be detailed later in the discussion topic. Laser-photobiomodulation was proposed and initiated on March 5^th^.

### 2.1. Materials and Methods

**Figure 3:**
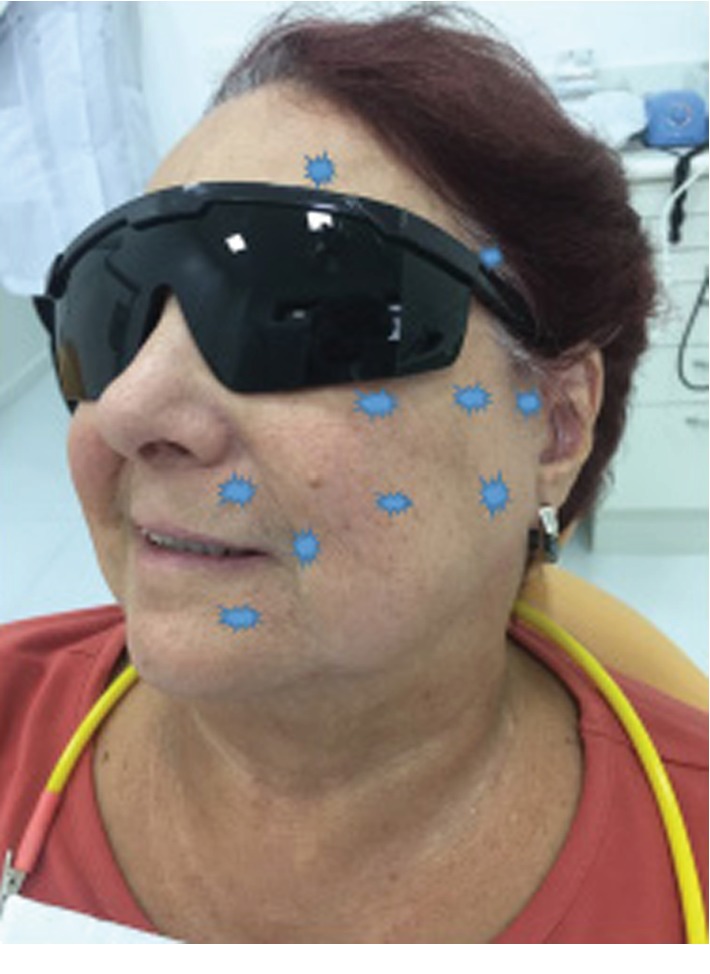
Laser application points.

A low-level laser (brand: DMC^™^) was previously measured to have a 100 mW output power density with a fiber diameter of 600 *μ*m. The protocol was punctual application of 3.3 J per point (120 J/cm^2^), 10 seconds each, in contact with the skin, infrared of 808 nm, and applied to ten points (Figure 3):

- Point 1: frontal muscle
- Point 2: temporal muscle
- Points 3, 4, and 5: zygomatic muscle
- Point 6: buccinator muscle
- Points 7: lip elevator
- Point 8: orbicularis of the lips
- Point 9: lip depressor
- Point 10: masseter

## 3. Results and Discussion

After the fifth session, the patient's recovery was remarkable, and she was both cooperative and satisfied. It should be pointed out that no other form of therapy was performed.

**Figure 4:**
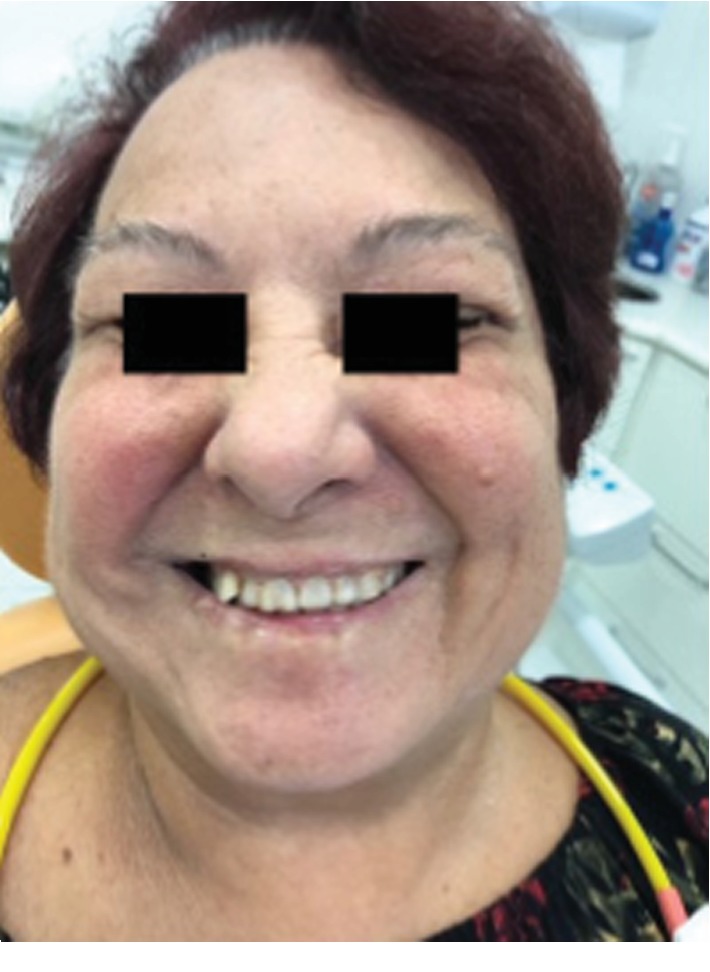
Results after ten sessions of L-PBM.

A total of 10 sessions were held, initially one session every 48 hours; after the fifth session, two weekly sessions were held until the remission of the condition. The treatment was ended on April 21^st^, 2018. Follow-up was performed every two weeks in May and June, followed by monthly evaluations to the present date, with no sequelae or recurrence (Figure 4). According to the HB Scale, the results indicate grade I (Table 1).

The results were evaluated according to the House-Brackmann Scale (1985).

Bell's palsy is generally a self-limiting condition, with the return of functions within a six-month period and with no substantial sequelae in most cases. However, a significant portion of patients have more severe conditions. It is estimated that 71% of affected individuals recover completely, about 13% only achieve partial recovery, and approximately 4% experience severe paralysis [3].

The conventional therapies include drug therapy (corticosteroids and antiviral drugs), facial exercises, massage, thermotherapy, electrical stimulation, acupuncture, and laser-photobiomodulation [1–4, 15].

Regarding the recovery time, the literature shows that, with conventional therapies, 69.5% of patients have good recovery after 3 months, while 30.5% have poor results [20].

Recovery results are often evaluated by the House-Brackmann Scale, assessing the levels of facial nerve injury and proposing the following order: normal, soft, moderate, moderate/severe, severe dysfunction, and complete paralysis [20, 21].

Some studies have demonstrated that L-PBM enhances the regeneration of neurons, with local and systemic effects, directly restoring nerve structure and their communication with the central nervous system. Some researchers have demonstrated that laser increases microcirculation, activating angiogenesis and stimulating nerve regeneration [2]. Laser-photobiomodulation stimulates photoreceptors present on the mitochondrial membrane, converting light energy into chemical energy, increasing ATP that enhances cellular functions and regeneration [2]. One of the possible explanations of the low-level laser effects is that it increases the activity of the enzymes involved in the mitochondrial respiratory chain, leading to an increase in ATP production, directly acting on the oxidative stress, that is increased in Bell's palsy [1, 5, 22]. Low-level lasers also have an anti-inflammatory effect, explained by the reduction of proinflammatory cytokines and increasing anti-inflammatory growth factors [22]. Once inflammation is one of the factors involved in the pathophysiology of facial palsy, the L-PBM can improve the restoration of the homeostasis of the tissues [2].

A recent study reports that oxidative stress is high among individuals with Bell's palsy [5]. As one of the important mechanisms of action of L-PBM is the restoration of normal intratissue oxygen levels, this could be an important therapeutic modality for both minimizing the occurrence of sequelae and accelerating complete recovery. While some studies have demonstrated such effects [2, 4, 12, 16, 22], there remains a need for the establishment of standardized protocols. The present case report demonstrates that laser-photobiomodulation, when used in the early stages, can be an effective treatment for Bell's palsy.

## 4. Conclusion

Idiopathic facial paralysis (Bell's palsy) is an event that has a benign course in the majority of cases but can leave sequelae and have negative social, professional, and psychological impacts. Apparently, the early institution of therapeutic measures improves the chances of a complete recovery. Laser-photobiomodulation is a promising treatment option in such cases. However, controlled clinical trials are needed to enable the establishment of safe, adequate, and individualized protocols.

## Figures and Tables

**Table 1:** House-Brackmann Scale evaluates the level of facial nerve paralysis.

Grade	Function level	Symmetry at rest	Eyes	Mouth	Forehead
I	Normal	Normal	Normal	Normal	Normal
II	Mild	Normal	Easy and complete closure	Slightly asymmetrical	Reasonable function
III	Moderate	Normal	With effort complete closure	Slightly affected with effort	Slight to moderate
IV	Moderately severe	Normal	Incomplete closure	Asymmetrical with maximum effort	None
V	Severe	Asymmetry	Incomplete closure	Minimal movement	None
VI	Total paralysis	Total paralysis	Total paralysis	Total paralysis	Total paralysis
